# Cytomegalovirus reactivation in a critically ill patient: a case report

**DOI:** 10.1186/s13256-018-1681-4

**Published:** 2018-06-11

**Authors:** Demet Demirkol, Umay Kavgacı, Burcu Babaoğlu, Serhan Tanju, Banu Oflaz Sözmen, Suda Tekin

**Affiliations:** 1 Istanbul University Istanbul Faculty of Medicine, Department of Pediatrics, Division of Pediatric Intensive Care, Istanbul, Turkey; 2 Koç University School of Medicine, Department of Pediatrics, Division of Pediatric Intensive Care, Istanbul, Turkey; 3 Koç University School of Medicine, Istanbul, Turkey; 4 Koç University School of Medicine, Department of Thoracic Surgery, Istanbul, Turkey; 5 Koç University School of Medicine, Department of Pediatrics, Division of Pediatric Hematology and Oncology, Istanbul, Turkey; 6 Koç University School of Medicine, Department of Infectious Diseases, Istanbul, Turkey

**Keywords:** CMV reactivation, Critically ill, Pediatric, Hemophagocytic lymphohistiocytosis, Sepsis, Multiple organ dysfunction

## Abstract

**Background:**

The aim of this case report is to discuss diagnostic workup and clinical management of cytomegalovirus reactivation in a critically ill immunocompetent pediatric patient.

**Case presentation:**

A 2-year-old white boy who had no medical history presented with respiratory distress and fever. His Pediatric Risk of Mortality and Pediatric Logistic Organ Dysfunction scores were 20 and 11, respectively. Our preliminary diagnosis was multiple organ dysfunction secondary to sepsis. Antibiotic treatment was started; he was intubated and artificially ventilated. Norepinephrine infusion was started. Hemophagocytic lymphohistiocytosis was diagnosed because our patient had elevated levels of serum ferritin, bicytopenia, splenomegaly, fever (> 38.5 °C), and hemophagocytosis shown in a bone marrow sample. Therapeutic plasma exchange and intravenously administered high-dose corticosteroid for hemophagocytic lymphohistiocytosis and continuous renal replacement treatment for acute renal failure were initiated. Following 5-day high-dose corticosteroid administration, therapeutic plasma exchange, and continuous renal replacement treatment, his clinical status and kidney and liver functions improved, and vasoactive requirement and ferritin levels decreased. He was extubated on the seventh day. On the tenth day of hospitalization he had a seizure and was diagnosed as having septic encephalopathy. His immune functions were found to be normal. Although his medical condition improved continuously, he had left spontaneous pneumothorax on the 21st day of admission as a complication of necrotizing pneumonia. Since pneumothorax persisted, left upper lobectomy surgery was performed on the 30th day of hospitalization. In the pathological examination of the excised lung tissue, features of cytomegalovirus infection were observed. Ganciclovir treatment was started. Serological tests indicated that our patient had cytomegalovirus reactivation. Antiviral treatment was stopped after 17 days, when cytomegalovirus deoxyribonucleic acid (DNA) polymerase chain reaction results became negative. He fully recovered and was discharged on the 50th day of admission.

**Conclusions:**

Cytomegalovirus reactivation in critically ill patients is a prevalent problem and shown to be associated with higher mortality and morbidity. In a case of serologic detection of cytomegalovirus reactivation without any clinical sign of infection, pre-emptive treatment could be considered with assessment of risks and benefits for each patient. Antiviral therapy is highly recommended for patients who have risk factors identified.

## Background

Cytomegalovirus (CMV) is one of the most prevalent viral pathogens, seropositivity ranging from 50 to 90% in immunocompetent individuals [1, 2]. Most primary CMV infections occur in the first decade of life presenting with nonspecific or subclinical symptoms and frequently resolve spontaneously. However, CMV can remain dormant in macrophages and monocytes, only to be reactivated when host immunity is compromised [3]. CMV reactivation in immunocompromised hosts is a well-known and studied problem as it leads to significant mortality and morbidity [4–6]. However, recent publications show that CMV reactivation in critically ill but previously immunocompetent hosts is also significantly prevalent, with an incidence as high as 15 to 20%, and could be associated to the clinical outcome of these patients [2, 7–9].

Conditions such as severe sepsis, extensive burns, multiorgan failure, and long-term use of corticosteroids might lead to a secondary immune deficiency, providing suitable host factors for CMV reactivation [3, 10]. These conditions may also give rise to acquired hemophagocytic lymphohistiocytosis (HLH), a disease that is characterized by abnormal cytokine production and excessive inflammation [11, 12]. Since HLH mimics many other conditions such as systemic infections, metabolic diseases, malignancies, and immunodeficiency, diagnosis is not easily established in clinical practice [13, 14]. An increased ferritin level above 500 ng/mL is seen in most cases, making it the first alarming finding that is suggestive of HLH. Current diagnostic criteria for HLH include: fever; splenomegaly; cytopenias; ferritin levels greater than 500 ng/mL; hypofibrinogenemia (< 150 mg/100 ml); and/or hypertriglyceridemia (fasting, > 265 mg/100 ml); low or absent natural killer (NK) cell activity; hemophagocytosis shown in bone marrow, spleen, or lymph nodes; and soluble interleukin-2 (IL-2) receptor (soluble CD25) levels greater than 2400 U/ml. Five out of these eight criteria is sufficient for HLH diagnosis [15–17]. HLH itself is also an immunosuppressive condition therefore it may result in various opportunistic infections and reactivation of dormant pathogens. CMV reactivation as a consequence of this acquired immunocompromised state could occur in patients with HLH. In this case report we describe a pediatric patient who had CMV reactivation after acquired HLH and our considerations regarding diagnostic workup and clinical management. To the best of our knowledge, this is the first report with sepsis, multiple organ failure, acquired HLH, and septic encephalopathy followed by CMV reactivation. The aim is to review the management of patients with CMV reactivation; the data for which are very limited and inconclusive.

## Case presentation

A 2-year-old white boy who had no medical history presented to University Hospital Emergency Department with respiratory distress and fever. He is the second child of a Turkish father and a 40-year-old Uzbek mother; his older sister has no health issues. He was born at term weighing 3350 g. Milestones were reached at the appropriate age.

On admission to the primary hospital, he had jaundice and confusion; his blood tests showed anemia, thrombocytopenia, and liver and kidney dysfunction. Suspecting malignancy, abdomen and thorax computed tomography (CT) and cranial magnetic resonance imaging (MRI) were obtained, which revealed bilateral pleural effusions with more severe parenchymal compression on his left lung. Periportal edema and hepatomegaly were detected in the abdominal CT. The cranial MRI was normal. On the day of admission, he was transferred to our pediatric intensive care unit (ICU).

At the time of admission, a physical examination revealed a confused boy (Glasgow Coma Scale was 13) with a length in 90th percentile and weight in 90th percentile. He had generalized edema and icterus. His pulse rate was 132 beats per minute (bpm), respiratory rate was 45 breaths per minute, blood pressure was 120/54 mmHg, and temperature was 35 °C. Respiratory sounds were attenuated in his left lung. His abdomen was distended; his liver was palpable 4 to 5 cm below ribs. His Pediatric Risk of Mortality and Pediatric Logistic Organ Dysfunction scores were 20 and 11, respectively. Our preliminary diagnosis was multiple organ dysfunction secondary to sepsis. Antibiotic treatments were started with vancomycin (60 mg/kg per day) and meropenem (120 mg/kg per day) after sampling of pleural effusion, which showed characteristics of empyema (Table 1). The cultures (urine, blood, catheter, and pleural fluid) were sterile. Due to increasing respiratory distress, he was intubated and artificially ventilated. A norepinephrine infusion was started to maintain cardiac output and prevent hypotension. HLH was diagnosed since he had elevated levels of serum ferritin, bicytopenia, splenomegaly, fever (> 38.5 °C), and hemophagocytosis shown in bone marrow sample, fulfilling five out of eight diagnostic criteria for acquired HLH. He had no urinary output for the last 72 hours; his blood urea nitrogen, uric acid, and creatinine levels were increased. Initial laboratory results are shown in Table 2. Therapeutic plasma exchange (TPE) and intravenously administered high-dose corticosteroid for HLH and continuous renal replacement treatment (CRRT) for acute renal failure were initiated.

**Table 1:** Pleural effusion sampling results

Quality	Turbid
Pleural glucose level (mg/dL)	16
Serum glucose level (mg/dL)	62
Pleural protein level (g/dL)	3.89
Serum protein level (g/dL)	3.96
Pleural lactate dehydrogenase level (U/L)	15,172
Serum lactate dehydrogenase level (U/L)	4503
Pleural leukocyte count (/mm^3^)	13,750

**Table 2:** Initial laboratory workup

	Patient’s result	Reference ranges
Hemogram		
Leukocyte count (/mm^3^)	7000	5500–15,500
Hemoglobin level (g/dL)	6	11.5–15.5
Platelet count (/mm^3^)	22,000	150,000–400,000
Coagulation parameters		
aPTT (seconds)	64	25–35
PT (seconds)	10	11–15
Fibrinogen (g/L)	7.11	1.25–3.0
Biochemistry panel		
Creatinine (mg/dL)	1.98	0.5–1.0
Uric acid (mg/dL)	9.8	2.2–6.6
BUN (mg/dL)	72	5–18
Ammonia (μmol/L)	26	17–68
Alanine aminotransferase (U/L)	65	5–45
Aspartate aminotransferase (U/L)	440	15–55
Alkaline phosphatase (U/L)	116	130–560
Gamma-glutamyl transferase (U/L)	13	5–24
Triglycerides (mg/dL)	132	31–108
Total protein (g/dL)	3.96	6.1–7.9
Albumin (mg/dL)	2.1	3.9–5.0
Direct bilirubin (mg/dL)	19.6	< 0.2
Total bilirubin (mg/dL)	19.9	0.3–1.2
C-reactive protein (mg/L)	322	0–5
Procalcitonin (μcg/L)	> 100	0–1
Lactate dehydrogenase (U/L)	4503	120–330
Ferritin (ng/mL)	10,699	7–140
Haptoglobin (mg/dL)	22	26–185

*aPTT* activated partial thromboplastin time, *BUN* blood urea nitrogen, *PT* prothrombin time

Following 5-day high-dose corticosteroid administration, TPE, and CRRT, his clinical status and kidney and liver functions improved, and his vasoactive requirement and ferritin levels decreased. The norepinephrine infusion was decreased gradually and stopped after 4 days. He was extubated on the seventh day. On the tenth day of hospitalization he had a seizure; electroencephalography, MRI, and lumbar puncture did not reveal pathological findings. Since we excluded other pathologies that may cause seizures, he was thought to have septic encephalopathy. Antiepileptic medication was started, and he did not have a seizure in the follow-up period. CRRT was continued intermittently, and completely stopped on the 14th day of hospitalization. The antibiotic treatments were stopped on the 14th day of treatment. His immune functions were also investigated; his lymphocyte subset panel and immunoglobulin levels were found to be normal (Table 3). The result of serologic testing for human immunodeficiency virus was negative.

**Table 3:** Immunological function tests

	Patient’s results	Reference range
Immunoglobulin levels		
IgM, serum (mg/dL)	30	41–164
IgA, serum (mg/dL)	68	14–122
IgE, total (IU/mL)	10	< 60
IgG, serum (mg/dL)	868	331–1164
Lymphocyte subset panel		
CD4+ lymphocyte (%)	43.3	23–48
CD4+ lymphocyte count (/μL)	1115.74	500–2400
CD8+ lymphocyte (%)	30.06	14–33
CD8+ lymphocyte count (/μL)	788.49	300–1600
CD 56+ cell (%)	12	4–23

*Ig* immunoglobulin, *CD* cell differentiation

**Table 4:** The patient’s cytomegalovirus serology

Serologic marker	Day 1	Day 35	Day 49
Cytomegalovirus IgG antibody (aU/mL)	–	85 (positive)	–
Cytomegalovirus IgG avidity	–	0.88 (index)	–
Cytomegalovirus IgM antibody	0.04 (negative)	0.41 (negative)	–
Cytomegalovirus DNA (PCR)	–	Positive	Negative
Cytomegalovirus concentration (copy/mL)	–	11306	–

*CMV* cytomegalovirus, *PCR* polymerase chain reaction, *DNA* deoxyribonucleic acid, *Ig* immunoglobulin

**Fig. 1:**
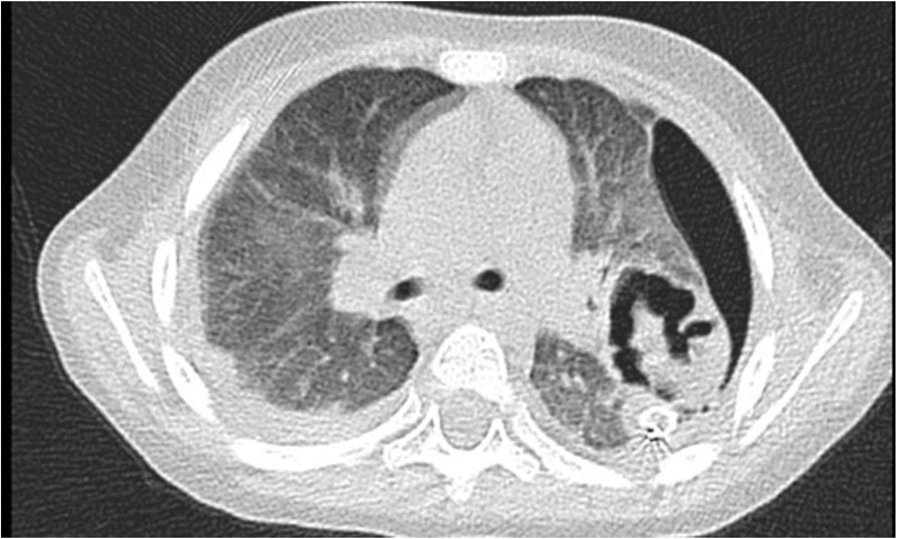
Axial computed tomography image of the patient before operation

Although his medical condition improved continuously, he had left spontaneous pneumothorax on the 21st day of admission as a complication of necrotizing pneumonia. Necrotizing pneumonia was diagnosed by thorax CT findings (Fig. 1). Since pneumothorax persisted, left upper lobectomy surgery was performed on the 30th day of hospitalization. In the pathological examination of the excised lung tissue, features of CMV infection were observed and ganciclovir (10 mg/kg per day) treatment was started 2 days after surgery. In order to confirm the presence of CMV infection, CMV deoxyribonucleic acid (DNA) polymerase chain reaction (PCR), CMV DNA IgG avidity index, and CMV IgG and IgM tests were utilized. CMV IgM was found to be negative; however, CMV IgG was positive with high CMV DNA IgG avidity index (0.88; Table 4). These results indicated that he had CMV reactivation, rather than an acute primary CMV infection. We determined the duration of ganciclovir treatment according to the level of CMV DNA PCR (10300 copies/mL) in the blood. Antiviral treatment was stopped after 17 days, when CMV DNA PCR results became negative. He fully recovered and was discharged from our hospital on the 50th day of admission. He had no health problem after discharge at 6-month follow-up at an out-patient clinic.

## Discussion

This case was challenging in many aspects such as having multiple serious diseases including empyema, sepsis, multiple organ failure, acquired HLH, and septic encephalopathy followed by CMV reactivation. The pathogen microorganism for lung infection leading to empyema was never identified, most probably because our patient had started antibiotic treatment days ago. At the time of admission he had renal, hepatic, respiratory, cardiac, and hematological dysfunction. Despite involvement of five or more organs, which is a negative prognostic factor in multiorgan failure cases, he recovered rather quickly after acquired HLH was controlled and organ functions were supported with TPE, high-dose steroid treatment, and CRRT.

An increased ferritin level in blood is strongly associated to HLH, and it may have a prognostic value regarding clinical outcome of the patient. A higher maximum ferritin level in the first 3 weeks of disease is found to be associated with higher mortality rates. Furthermore, the rate of decline of ferritin in response to treatment has also proved to be significant, as higher rate of decline (≥ 96% decrease) indicates a better prognosis than lower rate of decline (< 50% decrease) [18, 19]. After 5-day TPE and high-dose corticosteroid treatment, our patient’s ferritin levels decreased from 10,699 ng/mL to 1356 ng/mL (88% decrease) putting him into the average prognosis group.

Several studies claimed that CMV reactivation in previously immunocompetent critically ill patients causes a remarkable increase in overall mortality, duration of hospitalization, need for mechanical ventilation, and frequency of nosocomial infections [2, 20, 21]. Definite mechanisms for these negative effects are yet to be found, current hypotheses being inactivation of host’s immune defenses by CMV and cytopathic response caused by the overactive immune system [22]. Some researchers argued that CMV reactivation in critically ill patients could be merely an indicator of the patients’ poor clinical status rather than causing additional morbidity and mortality by its own effects [8]. However, meta-analyses indicated that when all the data are pooled together, CMV reactivation significantly affects patient outcome and increases mortality rates by up to 81% [20]. Current evidence demonstrates that CMV reactivation should be taken seriously, but still there is no consensus among medical professionals regarding the management of these patients. There is anecdotal data that showed timely initiation of TPE contributed to recovery from severe CMV infection [23]. In addition, clinical studies on CMV reactivation in critically ill previously immunocompetent patients are all conducted in adult populations and, as a result, the significance of CMV reactivation in the pediatric population with the same characteristics is not identified yet.

In our case, ganciclovir treatment was started as soon as pathological examination of the lung specimen revealed CMV infection. When we started antiviral treatment, CMV PCR DNA and CMV immunoglobulin (Ig)G and IgM levels were not reported yet, therefore differentiating primary infection from reactivation was not possible. Later, we concluded that our case had CMV reactivation, because CMV IgG avidity index was high (0.88) while CMV IgM antibody concentration was very low, and decided to continue antiviral treatment until CMV DNA PCR became negative.

There is no definitive management guideline on CMV reactivation in critically ill patients and for each individual case, the decision whether to start antiviral treatment or not, when to stop the treatment, and how appropriate dosing should be, is left to clinicians. Initiating ganciclovir treatment to all CMV seropositive patients (prophylaxis) was suggested in a couple of papers and it could potentially prevent CMV reactivation in critically ill patients. However, this approach would be far from ideal as ganciclovir has adverse effects like decreased creatinine clearance, anemia, thrombocytopenia, and neutropenia and may not be tolerable for every ICU patient, especially those with kidney dysfunction and hematological problems [24, 25]. Narrowing down the target patient group for prophylactic ganciclovir treatment could be achieved by testing every ICU patient for CMV seropositivity at admission. In cases of serologic detection of CMV reactivation without any clinical sign of infection, pre-emptive treatment could be considered with assessment of risks and benefits for each patient. Most authors advocated use of curative antiviral therapy in cases with proven CMV reactivation (PCR and/or serology) and presence of clinical signs of infection. Antiviral therapy, such as a regimen of ganciclovir 5 mg/kg per day for at least 2 weeks [22], is highly recommended for patients with primary lung infiltrates, impaired gas exchange, and a very high viral load (> 10,000/mL) concurrent with at least two risk factors such as: leukopenia, hemophagocytosis, absence of bacterial agent in cultures, mechanical ventilation duration of more than 2 weeks, increase in liver transaminase levels by 1.5-fold to threefold, and increase in bilirubin levels by 1.5-fold to threefold.

CMV reactivation in critically ill patients is a prevalent problem and shown to be associated with higher mortality and morbidity in numerous studies including meta-analyses. However, data regarding methods to accurately identify CMV reactivation, how to manage patients with CMV reactivation, and which patients should be treated, remain very limited and inconclusive. The prevalence of CMV reactivation in pediatric ICUs is not studied yet, and there is no current evidence that shows CMV reactivation affects children in the same manner as adults. Further research should be conducted to establish appropriate management guidelines for these patients.

## Conclusions

CMV reactivation in critically ill patients is a prevalent problem and shown to be associated with higher mortality and morbidity. In a case of serologic detection of CMV reactivation without any clinical sign of infection, pre-emptive treatment could be considered with assessment of risks and benefits for each patient. Antiviral therapy is highly recommended for patients who have risk factors identified.
